# Mono- and combinational drug therapies for global viral pandemic preparedness

**DOI:** 10.1016/j.isci.2022.104112

**Published:** 2022-03-17

**Authors:** Aleksandr Ianevski, Rouan Yao, Ronja M. Simonsen, Vegard Myhre, Erlend Ravlo, Gerda D. Kaynova, Eva Zusinaite, Judith M. White, Stephen J. Polyak, Valentyn Oksenych, Marc P. Windisch, Qiuwei Pan, Eglė Lastauskienė, Astra Vitkauskienė, Algimantas Matukevičius, Tanel Tenson, Magnar Bjørås, Denis E. Kainov

**Affiliations:** 1 Department of Clinical and Molecular Medicine (IKOM), Norwegian University of Science and Technology (NTNU), 7028 Trondheim, Norway; 2 Vilnius Ozo Gymnasium, Vilnius University, Vilnius 07171, Lithuania; 3 Institute of Technology, University of Tartu, 50411 Tartu, Estonia; 4 University of Virginia, Department of Cell Biology, Charlottesville, VA, USA; 5 Virology Division, Department of Laboratory Medicine and Pathology, University of Washington, Seattle, WA, USA; 6 Applied Molecular Virology Laboratory, Institut Pasteur Korea, 463-400 Gyeonggi-do, Korea; 7 Department of Gastroenterology and Hepatology, Erasmus MC-University, Medical Center, Rotterdam, Netherlands; 8 Department of Laboratory Medicine, Lithuanian University of Health Science, 44307 Kaunas, Lithuania; 9 Life Sciences Center, Vilnius University, 10257 Vilnius, Lithuania; 10 Institute for Molecular Medicine Finland, University of Helsinki, 00014 Helsinki, Finland

**Keywords:** Pharmaceutical preparation, Pharmaceutical science, Pharmacology, Chemistry

## Abstract

Broadly effective antiviral therapies must be developed to be ready for clinical trials, which should begin soon after the emergence of new life-threatening viruses. Here, we pave the way towards this goal by reviewing conserved druggable virus-host interactions, mechanisms of action, immunomodulatory properties of available broad-spectrum antivirals (BSAs), routes of BSA delivery, and interactions of BSAs with other antivirals. Based on the review, we concluded that the range of indications of BSAs can be expanded, and new pan- and cross-viral mono- and combinational therapies can be developed. We have also developed a new scoring algorithm that can help identify the most promising few of the thousands of potential BSAs and BSA-containing drug cocktails (BCCs) to prioritize their development during the critical period between the identification of a new virus and the development of virus-specific vaccines, drugs, and therapeutic antibodies.

## Graphical abstract

**Figure:**
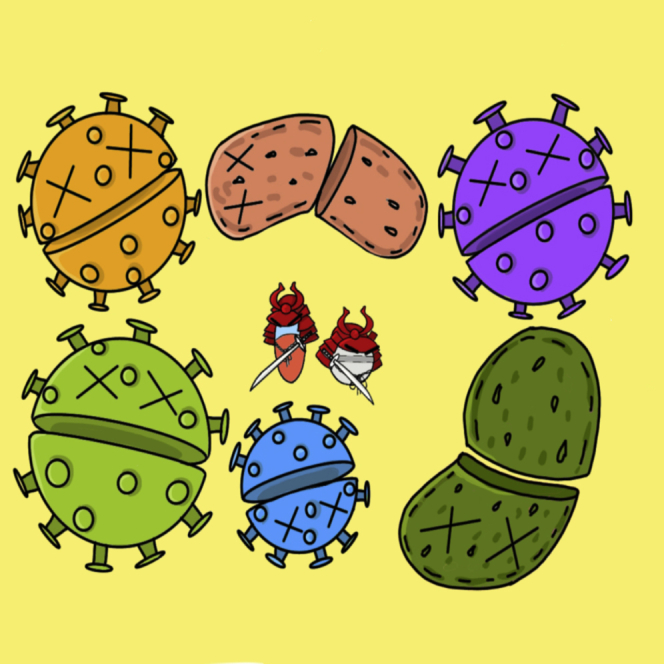

## Introduction

Despite advances in modern medicine, viral diseases consistently pose a substantial economic and public health burden throughout the world. In fact, both the World Health Organization and the United Nations have highlighted the specific need for better management of viral diseases as priorities for future development (World Health Organization, 2018). This burden is likely due to viruses’ ability to regularly emerge and re-emerge into the human population from natural reservoirs such as wild and domesticated animals, leading to unpredictable outbreaks and wildly destructive health consequences (Choi, 2021). However, despite this constant threat of viral outbreaks, the landscape of antiviral targets is still underdeveloped, with over 200 human viral diseases that lack approved antiviral treatments.

Because the development of novel antivirals is long, laborious, and often unprofitable, the current strategy for the management of viral outbreaks is heavily reliant on the development of vaccines over antiviral treatments (Monto, 2006). However, while vaccines are an effective public health measure to stop the community spread of a well-characterized virus, it is impossible to develop vaccines against viral diseases that may emerge in the future. Therefore, antiviral development remains a crucial aspect of viral disease management to ensure timely and effective treatment of infected individuals and to reduce virus transmission.

Antiviral drugs are approved medicines that stop viruses from multiplying. Currently, there are 179 approved antiviral drugs, which are derived from 88 unique drug structures. Antiviral drugs currently represent 4.4% of 4,051 approved medicines. However, 10 of 88 have been withdrawn due to side effects (Chaudhuri et al., 2018; Wishart et al., 2018). The most common side effects of many antiviral drugs are nausea, vomiting, allergic reactions, drowsiness, insomnia, heart problems, and dependence (Morris, 1994). Side effects can also be associated with the capacity of the drugs to either enhance or suppress intrinsic immune functions of infected cells or alter the activity of immune cells within the host (Holstein and McCarthy, 2017). Antiviral drugs with immunostimulatory properties could lead to “cytokine storm,” which could be associated with an overwhelming systemic inflammation that leads to multiple organ dysfunction and potentially death (Fajgenbaum and June, 2020). By contrast, antivirals with immunosuppressive properties can be beneficial for the treatment of “cytokine storm” (D'Elia et al., 2013). However, these drugs could prevent the development of adaptive immune responses allowing re-infections with the same or similar virus strains. Thus, antivirals without immunomodulatory properties are likely to be beneficial for the treatment of viral infections (Alijotas-Reig et al., 2020; Zusinaite et al., 2018).

Antiviral agents are molecules that have undergone pre-clinical development or clinical investigations against certain viruses but have not been approved for pharmaceutical use. Currently, there are thousands of antiviral agents in preclinical development and hundreds in clinical trials. It takes approximately 13–15 years and 2 billion USD to develop a new antiviral drug from an antiviral agent (Pizzorno et al., 2019).

Antiviral drugs and agents can be further divided into those that target the virus and those that target the host. Virus-directed antivirals target viral proteins, viral nucleic acids, or lipid envelopes. An example of a virus-directed antiviral is oseltamivir, an influenza drug that inhibits viral neuraminidase. Host-directed antivirals target cellular factors that mediate virus replication. In contrast to virus-directed antivirals, host-directed agents modulate the activity of host factors and pathways. An example of host-directed antiviral is maraviroc, an HIV-1 drug that targets the cellular CCR5 receptor to prevent a critical step in HIV-1 entry.

Antiviral drugs and agents come in numerous molecular forms including small molecules, peptides, neutralizing antibodies, interferons (IFNs), Crispr-Cas systems, si/shRNAs, and other nucleic acid polymers (NAPs) (Andersen et al., 2020; de Buhr and Lebbink, 2018; Levanova and Poranen, 2018; Lin and Young, 2014; Salazar et al., 2017; Vaillant, 2016). Of these, neutralizing antibodies, peptides, NAPs, and Crispr/Cas are mainly used as virus-directed interventions; IFNs are used as host-directed biologics, while small molecules can be either virus- or host-directed drugs.

Broad-spectrum antivirals (BSAs) can inhibit the replication of multiple viruses from the same or different viral families (Andersen et al., 2020). One efficient method of BSA development is drug repurposing/repositioning, a strategy for identifying new uses for approved or investigational antiviral drugs that are outside the scope of the original medical indication (Pushpakom et al., 2019). BSAs are cost-effective because the overall development cost can be distributed across many viral indications. Critically, robust BSA development fosters future pandemic preparedness because BSA activity facilitates enhanced coverage of newly emerged viruses.

Ongoing viral replication and prolonged exposure to certain drugs can lead to the selection of drug-resistant viruses through mutations in viral proteins. For example, mutations in HCV proteins confer resistance to NS3-4A, NS5A, and NS5B inhibitors (Ahmed and Felmlee, 2015). To mitigate the development of antiviral drug resistance, researchers developed antivirals that target protein-protein interactions rather than active sites of viral or host enzymes (Massari et al., 2021; Schormann et al., 2011). Another alternative is to combine antivirals (White et al., 2021). Additive, multiplicative, and synergistic drug combinations are more effective than monotherapies, allowing for successful treatments at lower dosage and reduction of harmful side effects. Indeed, a combination of IFN-a and ribavirin was the “gold standard” for the treatment of chronic HCV infection for more than a decade (Ilyas and Vierling, 2014). Furthermore, ribavirin- and IFN-a-containing combinations have been used against other viruses (Li et al., 2011; Tong et al., 2018) (NCT04412863), suggesting that BSA-containing combinations (BCCs) can be used to target a broad range of viruses.

Care needs to be taken when finding the correct BCCs. Drugs with unique mechanisms of action (MoA) are often paired together to minimize side effects and maximize efficacy. Drugs with the same MoA, such as nucleoside/nucleotide analogs, cannot be taken together (Zoulim, 2005) because they compete, rather than produce synergistic or additive effect. Such combinations could also have higher toxicity than monotherapies. In addition, drug antagonism can reduce the effectiveness of treatment and lead to an increased risk of virologic failure (failure to meet a specific drug target). Ideally, one wants the smallest number of drugs in cocktail due to the potential for increased toxicity and additional side effects with each additional drug (Radhakrishnan and Tidor, 2008).

Here we have reviewed the available scientific and clinical information and identified the basic principles behind activities of BSAs and BCCs to predict novel drug cocktails for the treatment of emerging and re-emerging viruses with pandemic potential. The approach described herein could facilitate the development of cost-effective and lifesaving countermeasures to fight new viral outbreaks.

## The landscape of broad-spectrum antiviral activities can be expanded

To identify known BSAs we have extensively reviewed published antivirals using PubMed.gov, ClinicalTrials.org and DrugBank.ca (Kim et al., 2021; Ursu et al., 2019; Wishart et al., 2018). Each of the resulting antiviral drug terms in this initial list was queried in combination with the terms “virus,” “antiviral,” or one of the known human viruses obtained from Virus Pathogen Database and Analysis Resource (Pickett et al., 2012). From this, we have compiled a list of antiviral drugs which we checked in the DrugBank. We desalted the compounds. Metals, mixtures, illicit and exclusively veterinary drugs were excluded. The returned results were examined to determine if antiviral activity has been demonstrated between the drug and two or more viruses from two different viral families. If antiviral activity could be established in more than 2 viral families, then all such drug-virus combinations would be recorded. Altogether, we identified 255 approved, investigational and experimental BSAs that target 104 human viruses from 24 families (Figure S1; Data S1).

Recently, we have tested several experimental, investigational, and approved BSAs against different viruses. We identified novel activities for saliphenylhalamide, gemcitabine, obatoclax, SNS-032, flavopiridol, nelfinavir, salinomycin, amodiaquine, obatoclax, emetine, homoharringtonine, atovaquone and ciclesonide, dalbavancin, vemurafenib, MK-2206, ezetimibe, azacitidine, cyclosporine, minocycline, ritonavir, oritavancin, cidofovir, dibucaine, azithromycin, gefitinib, minocycline, pirlindole ivermectin, brequinar, homoharringtonine, azacytidine, itraconazole, lopinavir, nitazoxanide, umifenovir, sertraline, amodiaquine and aripiprazole (Andersen et al., 2019; Bosl et al., 2019; Chen et al., 2020; Denisova et al., 2012, 2014; Herring et al., 2021; Ianevski et al., 2018, 2020c; Kakkola et al., 2013; Ko et al., 2021; Kuivanen et al., 2017; Li et al., 2020, 2021; Soderholm et al., 2016; Yang et al., 2021). These results suggest that the landscape of BSA activities is vast and that it can be further interrogated and expanded.

**Figure 1:**
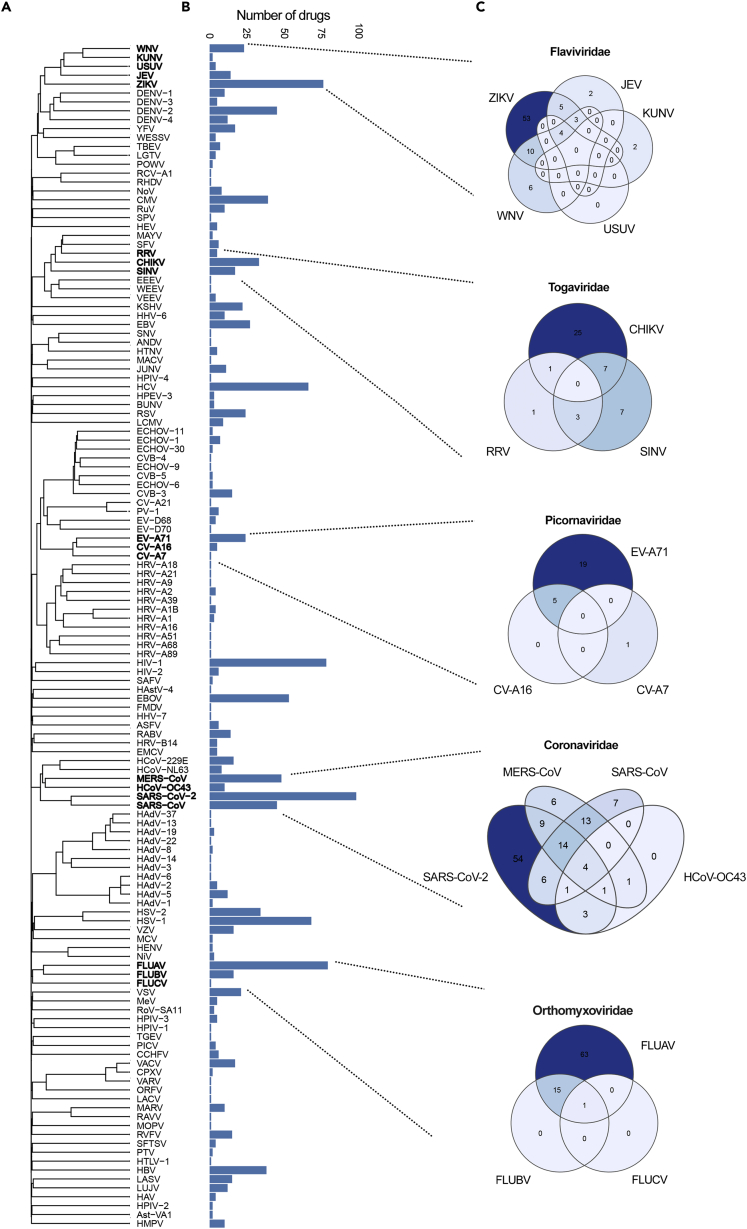
Drug activity-virus phylogeny relationship analysis (A) Phylogenetic tree of viruses constructed based on the amino acid sequences of viral pols and RTs. (B) Bar chart showing the number of BSAs active against the viruses shown in panel (A). (C) Venn diagrams showing the number of BSAs targeting closely related viruses.

To expand the activity spectrum of BSAs, we analyzed relationships between drug activity and virus phylogeny. For this, we first build a phylogenetic tree using the CLUSTALW2 algorithm and amino acid sequences of viral polymerases (pols) and reverse transcriptases (RTs) extracted from GenBank (Aiewsakun and Simmonds, 2018; Larkin et al., 2007) (Figure 1A). Notably, some viruses are represented by only small portions of pol and RT sequences (Data S1). Next, we identified the number of BSAs found to have activity against each corresponding virus (Figure 1B). Although the phylogenetically similar viruses will likely be responsive to the same drug, Figure 1C indicates that most BSAs are only tested against a small subpopulation of related viruses.

From this information, we can identify a wide range of previously untested BSA-virus interactions, which could demonstrate novel antiviral activities. For example, 54 BSAs have been proven effective for SARS-CoV-2, but not other coronaviruses indicating higher likelihood of antiviral activity between those 54 BSAs and several other coronavirus species. Due to the high probability of coronavirus emergence, this inference could further be applied to coronaviruses that may arise in the future. However, it is important to note that our analysis is limited to viruses that encode their own pols or RTs and for which full- or near full-length sequences of these enzymes are available. For viruses that do not encode their own pols and RTs or are thus far poorly characterized, an analysis of virus taxonomy and BSA activity may be required to make similar inferences (Kuhn, 2021).

## Structure-activity relationship analysis identifies novel broad-spectrum antiviral candidates

**Figure 2:**
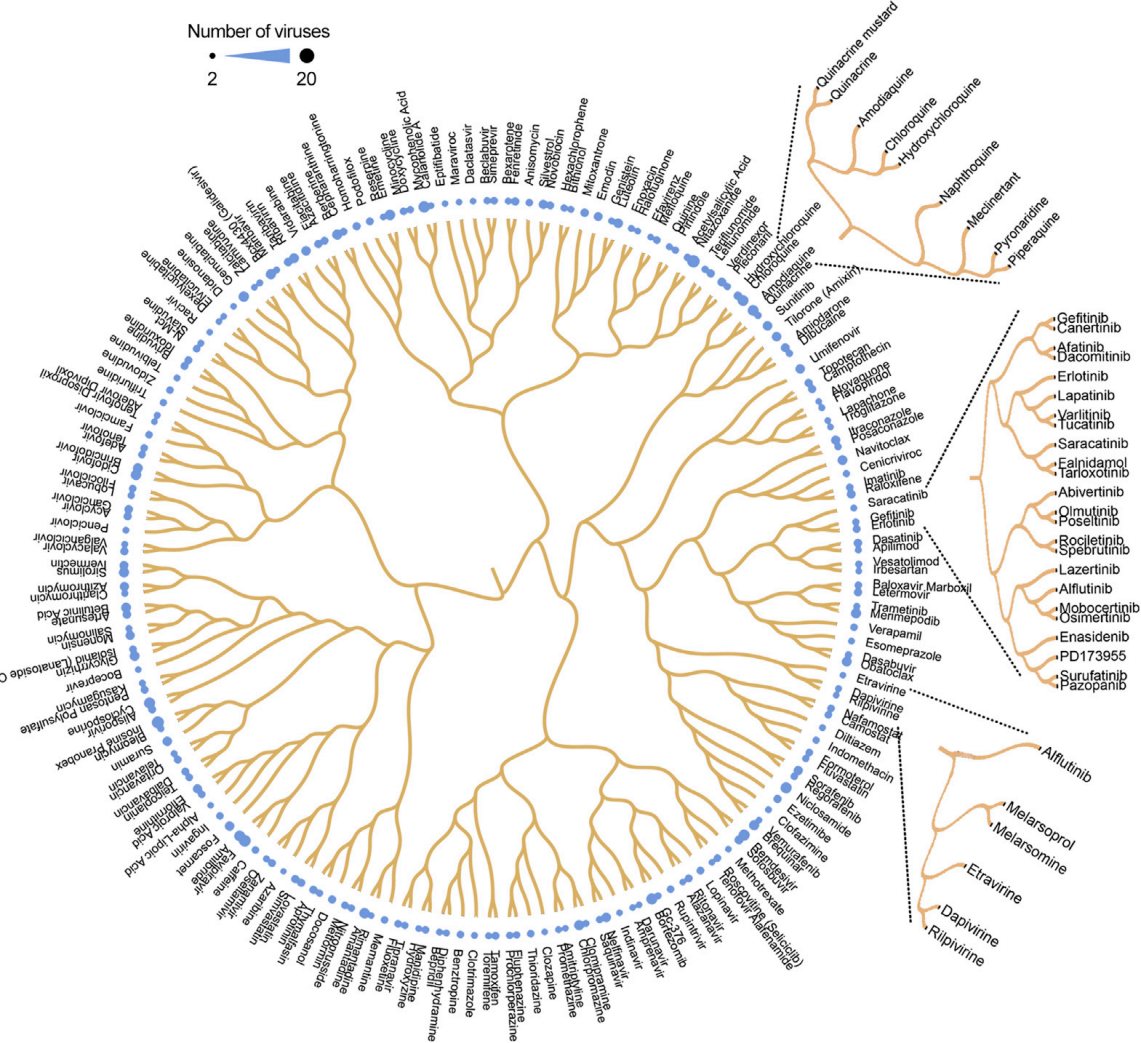
Structure-activity relationship analysis identifies compounds structurally similar to known BSAs The circular dendrogram shows the SAR of BSAs from our database. We also used SAR analysis to identify BSA candidates from the list of 11,834 compounds from DrugBank. Three compound sub-clusters that include two or more know BSAs are shown.

To expand the list of potential BSAs, we performed a drug structure–activity relationship (SAR) analysis of 11,834 compounds from DrugBank (Wishart et al., 2018), including 255 BSAs. The compound structures were obtained in the form of SMILES from the PubChem database (Kim et al., 2021). We used the most popular method, extended connectivity fingerprints of diameter 4 (ECFP4), to calculate the structural similarity of compounds (Rogers and Hahn, 2010). We clustered compounds based on their structural similarities and extracted the compound sub-clusters that include two or more BSAs (Data S1). Three such sub-clusters are shown in Figure 2. From this analysis, we can propose several new candidates for investigation as BSAs based on their structural similarities to existing BSAs. For example, the drugs domiphen, bephenium, pranlukast, afimoxifene, ospemifene, and fispemifene share structural similarities with tamoxifen and toremifene, known BSAs with activity against filoviruses and coronaviruses (Martin and Cheng, 2020; Montoya and Krysan, 2018; Tummino et al., 2021; Zhao et al., 2016). Based on structural similarity alone, we can identify these drugs as likely candidates for BSA activity. Similarly, pyronaridine, naphthoquine, meclinertant, and piperaquine are clustered together with BSAs quinacrine, amodiaquine, chloroquine, and hydroxychloroquine (Kaur and Kumar, 2021); melarsomine, melarsoprol, and FF-10101-01 are clustered together with BSAs etravirine, dapivirine, and rilpivirine (De Clercq, 2005); and CUDC-101, lapatinib, varlitinib, tucatinib, PD-168393, CP-724714, AZD-0424, tarloxotinib, canertinib, afatinib, dacomitinib, AV-412, falnidamol, enasidenib, LY-3200882, HM-43239, PD173955, PD-166326, abivertinib, olmutinib, poseltinib, spebrutinib, rociletinib, lazertinib, mobocertinib, osimertinib, alflutinib, and TOP-1288 are clustered with BSAs erlotinib, saracatinib, and gefitinib (Schor and Einav, 2018). Thus, we demonstrate that this type of SAR analysis could identify critical BSA scaffolds and predict novel BSAs.

## Virus and host targets for broad-spectrum antivirals

**Figure 3:**
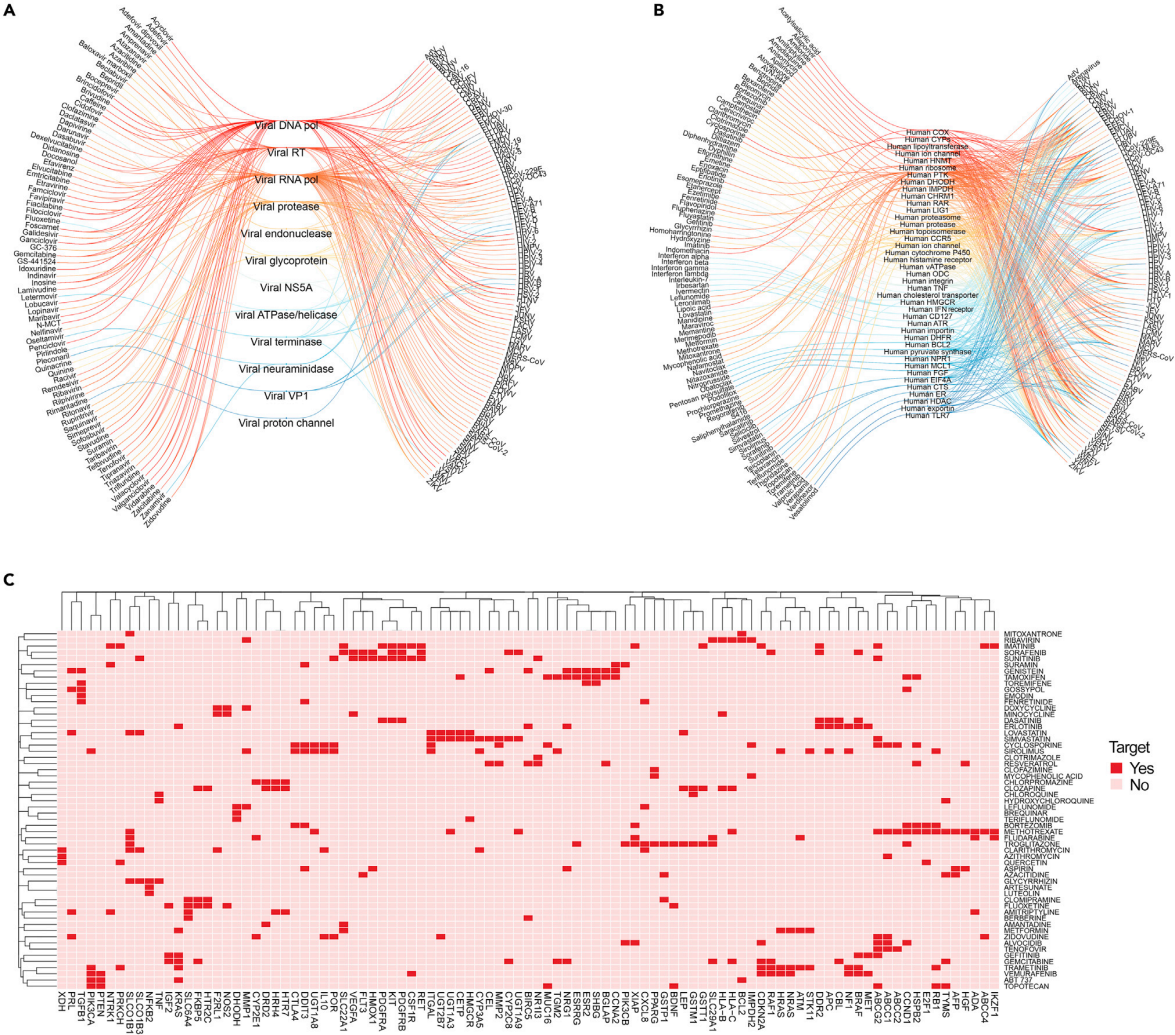
Virus and host targets for BSAs (A) Eye diagram showing virus-directed BSAs linked to viruses through potential targets. (B) Eye diagram showing host-directed BSAs linked to viruses through potential targets. (C) Common targets of 58 BSAs, which possess immunomodulatory properties. Targets with interaction group scores <0.05 as well as unique targets were omitted. Clustering was performed to show highlight targets for BSAs.

We next reviewed the known or suspected primary BSA targets (Data S1). We were able to identify primary targets for a fraction of BSAs. Most virus-directed BSAs work by inhibiting viral nucleic acid synthesis or protein processing (Figure 3A). Among host-directed BSAs, mechanisms appeared to be more varied and included the inhibition of protein translation, trafficking, modification, or degradation, receptor-mediated signaling, lipid metabolism, etc. (Figure 3B). However, in contrast to most host-directed BSAs that work through the inhibition of host factors, several host-directed BSAs also work to activate innate immune responses against viruses. For example, IFNs are natural host-directed activators that bind their receptors to trigger cellular antiviral responses, which attenuate viral replication (Park and Iwasaki, 2020), and ABT-263 (navitoclax) targets the Bcl-xL protein to initiate apoptosis of infected cells without affecting non-infected cells (Ianevski et al., 2020a). Some BSAs, such as suramin, can simultaneously target host and viral factors (Langendries et al., 2021; Yin et al., 2021). Lastly, some BSAs are given in the form of prodrugs such as ganciclovir and gemcitabine which are activated by virus or host factors to achieve their antiviral effect (Walther et al., 2017).

By interrogating the Drug-Gene Interaction database (Fajgenbaum and June, 2020), we found that several host-directed BSAs can target multiple cellular factors involved at several stages of viral replication. These extra drug targets are generally unexplored and are most likely associated with side effects, although they may also affect viral replication. Perhaps, the most important of these targets are immunomodulatory. We compiled the BSAs with secondary immunomodulatory targets and showed those in Figure 3C. From this, we found that many BSAs target similar clusters of immunomodulatory genes, indicating some structural and functional similarities between the targets, but no overarching immunomodulatory targets that may suggest a contribution to antiviral activity. Further analysis is needed to elucidate the exact role that these immunomodulatory targets play in host pharmacodynamics or their contribution to antiviral activity. Notably, the potential immunomodulatory side effects of BSAs can be mitigated by lower dosage of drugs in synergistic combinations.

## Broad-spectrum antiviral-containing drug combinations for the treatment of viral infections

Despite demonstrated efficacy at the early stages of drug development, many antiviral monotherapies are often found to be ineffective in clinical settings (Consortium et al., 2021). Because of this, antiviral cocktails have increasingly become the focus of drug developers. Antiviral combinations have several benefits over monotherapies. Namely, they can prevent the development of drug-resistant strains by completely halting viral replication, an advantage rarely achieved with monotherapies. Further, drugs administered together as cocktails may achieve an expanded antiviral activity, allowing for the treatment of multiple types of viral infections at once (Shyr et al., 2021). Because of this, BCCs are favorable candidates for front-line therapy against poorly characterized emerging viruses, re-emerging drug-resistant viral variants, and viral co-infections.

Indeed, BCCs have become a standard treatment of rapidly evolving viruses, such as HIV and HCV (www.drugs.com/drug-class/antiviral-combinations.html). These include triple and quadruple drug combinations such as abacavir/dolutegravir/lamivudine (Triumeq), darunavir/cobicistat/emtricitabine/tenofovir (Symtuza), ledipasvir/sofosbuvir (Harvoni), sofosbuvir/velpatasvir (Epclusa), and lopinavir/ritonavir (Kaletra). Furthermore, many dual drug combinations are now in clinical trials against SARS-CoV-2, HCV, HBV, HSV-1, and other viral infections (Ianevski et al., 2020b). In addition, many BCCs have been tested *in vitro* and in animal models (Dyall et al., 2018; Finch et al., 2021; Herring et al., 2021; Ianevski et al., 2020c, 2021a, 2021b) (Li et al.). These and other studies further demonstrate the potential for antiviral combinations for the treatment of emerging and re-emerging viral infections.

**Figure 4:**
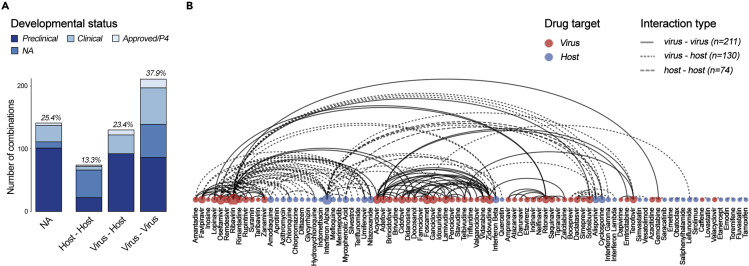
Drug-target interactions in BSA-containing combinations (A) Developmental statuses and targets of BCCs. (B) Examples of BCCs targeting virus, host, or both factors. A random walks algorithm was used to group the drug combinations based on their targets (Wang et al., 2020).

To underscore the potential benefits and provide an organized summary of known dual antiviral drug combinations, we manually reviewed scientific literature and patent applications and constructed a BCC database (Data S1). The database comprises 538 drug cocktails. It includes 612 unique drugs and covers 68 viruses. We were able to identify primary targets for 415 drugs (Figure 4). Of these, we found that 211 BCCs have components that both primarily target viral factors, 74 have components that both primarily target host factors, and 130 BCCs in which one drug primarily targets the virus while the other primarily targets the host. We were not able to identify specific targets for 160 BCCs due to one or more BSA in the BCC having an unknown mechanism of action. We suspect that the overrepresentation of virus-virus and virus-host targeting BCCs is as drugs that were developed to specifically target virus factors may be more successful in achieving a direct antiviral effect while minimizing severe side effects. Thus, virus-virus and virus-host targeting BCCs are superior to host-host BCCs in many ways, including the leveraging of antiviral synergism, reduction of toxicity. However, host-host targeting BCCs have lower risk of drug resistance and an expanded spectrum of antiviral activity.

## Association between infected organ systems and routes of broad-spectrum antivirals/BCCs administration

**Figure 5:**
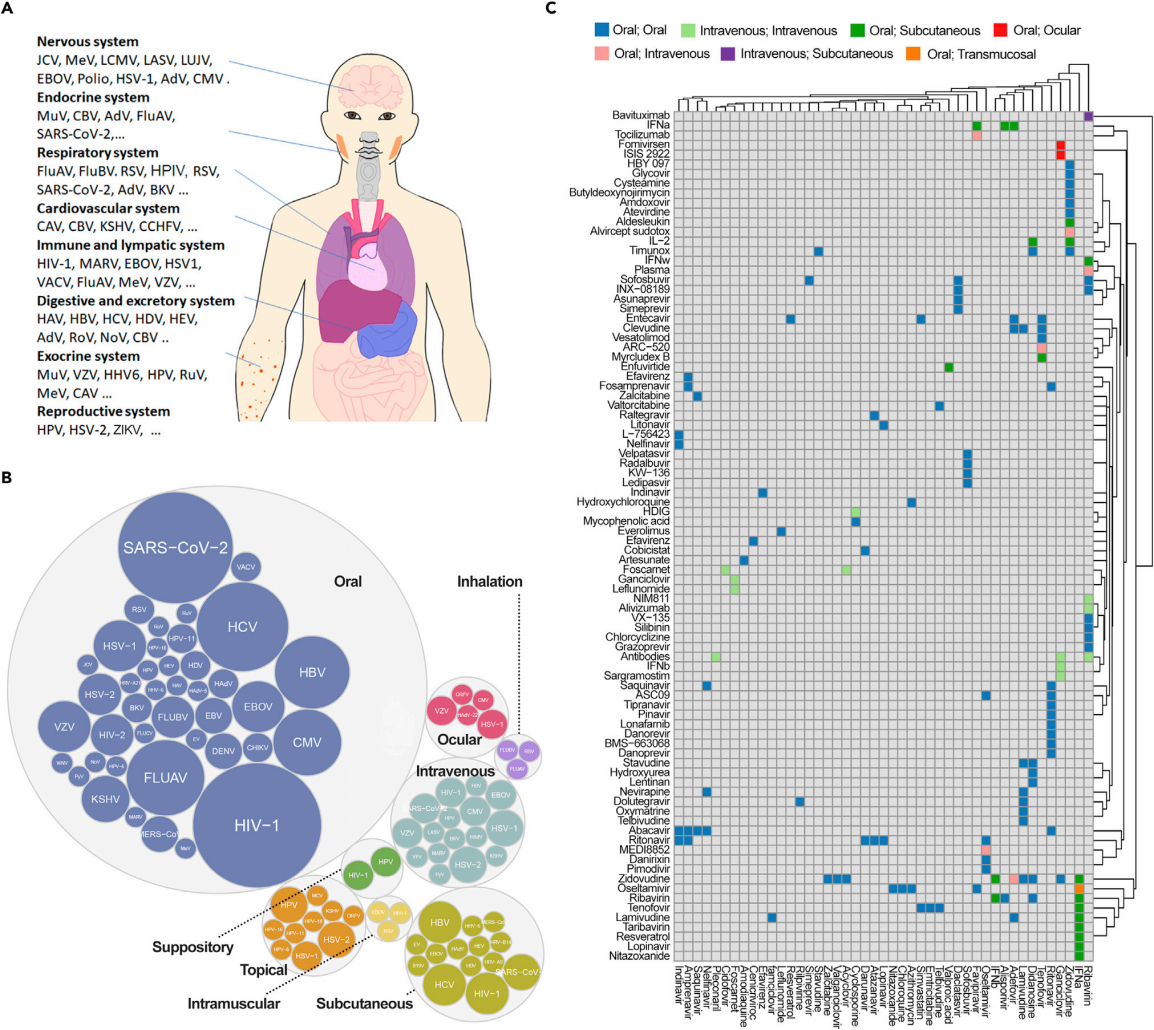
Routes of administration (RoA) of BSAs and BCCs (A) Organ systems that are preferentially affected by different viruses. (B) RoA of BSAs. Sizes of the colored bubbles reflect the number of BSAs developed against a certain virus. (C) RoA of BCCs. Colored squares indicate the combined RoA of drugs in BCCs. Gray shading indicates that antiviral activity has either not been studied or reported for the drug combination in question. Data S1. Broad-spectrum antivirals (BSAs), their targets, mechanisms of action, immunomodulatory properties, routes of delivery, BSA-containing drug combinations (BCCs), and BSA and BCC scores.

Viruses often preferentially infect hosts in one or more specific organ systems of the human body (Figure 5A). In theory, BSAs and BCCs must be rapidly delivered to the infected organs using an amenable route of administration (RoA) to preserve the drug structure, maximize antiviral effect, and reduce drug toxicity or other adverse events. For example, if a virus infects and replicates in the respiratory system, medications administered by inhalation may be preferable. Likewise, if the virus infects the cardiovascular system, intravenous drug administration could be considered, etc. However, intravenous administration prevents widespread use of the BCCs because use is restricted to specialized care centers such as hospitals. In cases of advanced or systemic virus infections that affect multiple organ systems, antivirals intravenous administration may be preferable. However, most of the BSAs and BCCs reviewed here are delivered orally, most likely due to the preferential development of orally bioavailable drugs by pharmaceutical companies because of their increased marketability and potential for global distribution (Figures 5B and 5C).

## Broad-spectrum antiviral and BCC scoring systems

To identify the most promising monotherapies we developed a six-component BSA scoring system:

1) SAR component (C_SAR_):
  • if the BSA is identical to a drug that has been developed or is currently under development for the virus of interest (voi), C_SAR_ = 1;
  • if the BSA is structurally similar to a drug that was developed or under development against the voi, C_SAR_ = 0.5;
  • if the BSA has a distinct structure, C_SAR_ = 0;
2) Drug developmental status component (C_DDS_; only applies to BSAs for which C_SAR_ = 1):
  • if the BSA is approved or is in phase 4 clinical trials against the Voi, C_DDS_ = 1;
  • If the BSA is in phase 1-3 clinical trials, C_DDS_ = 0.75;
  • if the BSA has been tested *in vivo*, C_DDS_ = 0.5;
  • if the BSA has been tested *in vitro*, C_DDS_ = 0.25;
  - if the BSA has not been tested, C_DDS_ = 0;
3) Drug target relevance component (C_TR_):
  • if the confirmed primary target of the BSA in question is associated with Voi replication (the drug target is essential for Voi replication), C_TR_ = 1;
  • if not, C_TR_ = 0;
4) Drug immunomodulatory component (C_IC_):
  • if the BSA does not interfere with host immune response, C_IC_ = 1;
  • if the BSA is immunomodulatory, C_IC_ = 0;
5) Drug RoA component (C_RoA_):
  • if the RoA of the BSA is well-suited for the diseased system (e.g., inhalation of drug for the treatment of respiratory viruses), C_RoA_ = 1;
  • if not, C_RoA_ = 0;
6) Phylogeny component (C_Phyl_):
  • if the Voi is in the same genus as the virus for which the BSA has been developed, C_Phyl_ = 1;
  • if the Voi is in the same family, C_Phyl_ = 0.5;
  • if the Voi is in a closely related family, C_Phyl_ = 0.25;
  • if the Voi is distantly related, C_Phyl_ = 0.

To calculate the final BSA score, we sum the points across all six components using the following formula:

(Equation 1) BSA score=CSAR+CDDS+CTR+CIC+CRoA+CPhyl

For example, the BSA score of favipiravir in relation to its activity against Ebola virus (EBOV) is 5.57. Favipiravir is an orally available nucleoside analog, which blocks viral RNA synthesis by inhibiting viral RdRP activity. Its immunomodulatory properties were not reported, and it is in phase 3 clinical trials against EBOV (NCT02329054). Therefore, the component values are as follows: C_SAR_ = 1, C_DDS_ = 0.75, C_DTR_ = 1, C_IC_ = 1, C_RoA_ = 1, and C_Phyl_ = 1 (Data S1).

Another example is merimepodib. Its BSA score in relation to its EBOV activity is 4.25. Merimepodib is an orally available inhibitor of host inosine monophosphate dehydrogenase (IMPDH), which controls the intracellular guanine nucleotide levels that are required for viral RNA synthesis. It possesses anti-EBOV activity *in vitro* and suppresses host immunity (Jain et al., 2001; Tong et al., 2018). Therefore, the component values are as follows: C_SAR_ = 1, C_DDS_ = 0.25, C_DTR_ = 1, C_IC_ = 0, C_RoA_ = 1, and C_Phyl_ = 1 (Data S1).

To identify the most promising combinational therapies, we invented a four-coefficient BCC scoring system. It utilizes the following BCC coefficients:

1) Drug interaction coefficient (k_DI_):
  • if the MoAs for each component of the combination are different, k_DI_ = 1;
  • if the MoAs are the same (for example, if both components are nucleoside analogs), k_DI_ = 0.5;
2) Drug-target interaction coefficient (k_DTI_):
  • if both BSA components target viral factors (the combination for which minimum side effects are expected), k_DTI_ = 1.2;
  • if one BSA targets a viral factor and one BSA targets a host factor, k_DTI_ = 1.1;
  • if both BSA components target host factors (the combination for which maximum side effects are expected, k_DTI_ = 1;
3) Drug-targeted stage of replication cycle coefficient (k_DRS_):
  • if both BSA components target the same stage of the virus life cycle (entry, viral replication, or exit), k_DRS_ = 1.2;
  • if the BSA components target different stages of the viral life cycle, k_DRS_ = 1;
4) Drug RoA coefficient (k_RoA_):
  • if both BSA components can be administrated by the same route and if the RoA can be used for targeted delivery to the diseased system, k_RoA_ = 1.2;
  • if both BSA components can be administrated by the same route, but the RoA cannot be used for targeted delivery to the diseased system, k_RoA_ = 1;
  • if the two BSA components cannot be administrated via the same route, k_RoA_ = 0.8;

From these we calculate a BCC score using the following formula:

(Equation 2) BCCscore=kDI∗kDTI∗kDRS∗kRoA∗(BSAscoredrug1+BSAscoredrug2)

If the BCC score exceeds the sum of the individual BSA scores by 5, we consider this combination to be effective (Data S1). For example, for favipiravir-merimepodib targeting EBOV, the k_DI_ is 1.0 because the MoAs of the drugs are different; the k_DTI_ is 1.1, because the drugs target viral RdRP and host IMPDH; the k_DRS_ is 1.2, because both drugs reduce the synthesis of viral RNA, and k_RoA_ 1.2, because both drugs can be taken orally, which allows delivery of the combination to multiple infected organs. Therefore, the BCC score of favipiravir-merimepodib is 15.8, whereas the combined BSA score of the combination is 10 (Table 1). Because the BCC score is greater than the combined BSA score by over 5 points, this combination would be considered to have high potential based on our scoring system. Indeed, by reviewing the literature, we found that this combination has been independently tested against EBOV *in vitro* and was shown to be effective (Tong et al., 2018).

**Table 1:** Examples of published and predicted BCCs, for which BCC scores exceed the sum of the individual BSA scores by 5

Virus	Family	Case fat. rate, %	Infected system	Drug 1 Drug 2	Sum of BSA scores	BCC score	Reference
Published BCCs							
EBOV	Filoviridae	66	Multiple	Favipiravir Merimepodib	10.0	15.8	(Tong et al., 2018)
LASV	Arenaviridae	13	Multiple	Ribavirin Merimepodib	9.0	14.3	(Tong et al., 2018)
HIV-1	Retroviridae	47	Multiple	Amprenavir Efavirenz	12	17.3	(Falloon et al., 2000)
				Efavirenz Indinavir	12	17.3	NCT00002387
FLUAV	Orthomyxoviridae	0.003	Respiratory system	Favipiravir Pimodivir	11.8	16.9	(Byrn et al., 2015)
				IFN-a Ribavirin	9.5	15.1	NCT01146535
HBV	Hepadnaviridae	40	Multiple	Telbivudine Alisporivir	9.3	14.7	(Phillips et al., 2015)
HCV	Flaviviridae	6.3	Multiple	Daclatasvir Sofosbuvir	12	17.3	NCT03200184
				Daclatasvir Simeprevir	12	17.3	NCT01628692
				Mericitabine IFN-a	10.8	17.0	(Wedemeyer et al., 2013)
				Ribavirin IFN-a	10	15.8	(Bellobuono et al., 1997)
ZIKV	Flaviviridae	n.a	Multiple	Favipiravir IFN-a	9.5	15.05	(Pires de Mello et al., 2018)

**Predicted BCCs**							

EBOV	Filoviridae	66	Multiple	IFN-b N4-hydroxycytidine	10.3	16.2	
				Favipiravir Tilorone	10.8	17.0	
MARV	Filoviridae	50	Multiple	Favipiravir Tilorone	10.5	16.6	
LUJV	Arenaviridae	80	Multiple	Favipiravir AVN-944	10.5	16.6	
				Favipiravir Brequinar	9.5	15.1	
				Ribavirin Merimepodib	8.5	13.5	
				Ribavirin AVN-944	10.0	15.8	
JUNV	Arenaviridae	25	Multiple	Favipiravir Merimepodib	9.8	15.4	
				Favipiravir Caffeine	10.8	17.0	
LASV	Arenaviridae	13	Multiple	Favipiravir Merimepodib	9.8	15.4	
HTNV	Hantaviridae	7	Multiple	Baloxavir Zidovudine	10.5	16.6	
				Baloxavir Favipiravir	10.5	16.6	
ANDV	Hantaviridae	23	Multiple	Baloxavir Favipiravir	9.0	14.3	
SNV	Hantaviridae	50	Multiple	Baloxavir Favipiravir	9.3	16.0	
LACV	Peribunyaviridae	1	Multiple	Baloxavir Favipiravir	8.5	14.7	
PTV	Phenuiviridae	n.a	Multiple	Baloxavir Favipiravir	8	13.8	
SFTSV	Phenuiviridae	21	Multiple	Baloxavir Favipiravir	11.0	17.4	
CCHFV	Nairoviridae	25	Multiple	Favipiravir Baloxavir	9.0	15.6	
FLUAV	Orthomyxoviridae	0.003	Respiratory system	Baloxavir Pimodivir	11.8	16.9	
VZV	Herpesviridae	0.1	Multiple	Foscarnet Favipiravir	8.5	16.2	
				Foscarnet Remdesivir	8.5	14.7	
				Foscarnet Sofosbuvir	8.5	14.7	
				Foscarnet Taribavirin	8.5	14.7	
				Foscarnet Ribavirin	7.5	13.0	
HTLV-1	Retroviridae	N/A	Multiple	Etravirine Emtricitabine	7.0	12.1	
				Didanosine Etravirine	7.0	12.1	
				Zalcitabine Etravirine	7.0	12.1	
HIV-1	Retroviridae	47	Multiple	Didanosine Rilpivirine	12.0	20.7	
				Etravirine Emtricitabine	12.0	20.7	
				Atazanavir Rilpivirine	12.0	17.3	
				Etravirine Adefovir	10.8	18.6	
				Rilpivirine Racivir	10.8	18.6	
HBV	Hepadnaviridae	40	Multiple	Nitazoxanide Valacyclovir	9.0	14.3	
				Nitazoxanide Zalcitabine	8.3	13.1	
NoV	Caliciviridae	n.a	Digest. system	Beclabuvir Mycophenolic acid	9.5	15.1	
DENV	Flaviviridae	0.37	Multiple	Brequinar GS-441524	9.5	15.05	
				Brequinar Azauridine	9.5	15.05	
				GS-441524 IFN-a	9.5	15.05	
				Azauridine IFN-a	9.5	15.05	
HCV	Flaviviridae	6.3	Multiple	Sofosbuvir IFN-a	11	17.4	
				INX-08189 IFN-a	10.3	16.2	
				Mericitabine Mycophenolic acid	10	15.8	
				RibavirinMycophenolic acid	9.3	14.7	
				Sofosbuvir Mycophenolic acid	10.3	16.2	
				INX-08189 Mycophenolic acid	9.5	15.1	
				Boceprevir Mericitabine	12	17.3	
				Sofosbuvir Boceprevir	12	17.3	
				Simeprevir Boceprevir	12	17.3	
				Simeprevir Mericitabine	12	17.3	
				Simeprevir Sofosbuvir	12	17.3	
				Daclatasvir Mericitabine	11.8	16.9	
				Daclatasvir Boceprevir	12	17.3	
ZIKV	Flaviviridae	n.a	Multiple	Clofazimine IFN-a	9.5	15.1	
				Rilpivirine Teriflunomide	9.8	15.4	
				Rilpivirine Mycophenolic acid	9.8	15.4	
				Rilpivirine IFN-a	9.8	15.4	
				Rilpivirine Brequinar	9.8	15.4	
				Rilpivirine Merimepodib	9.8	15.4	
				Clofazimine Mycophenolic acid	9.5	15.1	
				Clofazimine Teriflunomide	9.5	15.1	
				Clofazimine Brequinar	9.5	15.1	
				Clofazimine Merimepodib	9.5	15.1	
				Favipiravir Mycophenolic acid	9.5	15.1	
				Favipiravir Teriflunomide	9.5	15.1	
				Favipiravir Brequinar	9.5	15.1	
				Favipiravir Merimepodib	9.5	15.1	
				Azaribine IFN-a	9.5	15.1	
				Azaribine Mycophenolic acid	9.5	15.1	
				Azaribine Teriflunomide	9.5	15.1	
				Azaribine Brequinar	9.5	15.1	
				Azaribine Merimepodib	9.5	15.1	
				Efavirenz IFN-a	9.5	15.1	
				Efavirenz Mycophenolic acid	9.5	15.1	
				Efavirenz Teriflunomide	9.5	15.1	
				Efavirenz Brequinar	9.5	15.1	
				Efavirenz Merimepodib	9.5	15.1	
				Gemcitabine IFN-a	8.5	13.5	
				Ribavirin IFN-a	8.5	13.5	
				Ribavirin Mycophenolic acid	8.5	13.5	
				Ribavirin Teriflunomide	8.5	13.5	
				Ribavirin Brequinar	8.5	13.5	
				Ribavirin Merimepodib	8.5	13.5	
				Sofosbuvir IFN-a	10.8	17.0	
				Sofosbuvir Mycophenolic acid	10.8	17.0	
				Sofosbuvir Teriflunomide	10.8	17.0	
				Sofosbuvir Brequinar	10.8	17.0	
				Sofosbuvir Merimepodib	10.8	17.0	

In contrast, the BCC score for favipiravir-ribavirin against EBOV is 7.3. This is lower than the sum of individual BSA scores of favipiravir and ribavirin, which is 8.5, which predicts suboptimal performance for this combination (Table 1). Literature review shows that this prediction is consistent with efficacy studies in monkeys (Madelain et al., 2020). Thus, we demonstrated that the results from our scoring system are consistent with real-life experimental evidence.

Next, we used our scoring system for the identification of novel potential BCCs (Data S1). As mentioned above, we focused on novel combinations for which BCC scores exceed the sum of the individual BSA scores by > 5. In this way, we have identified several unexplored drug combinations that may be prioritized for development in preparation for future resurgent outbreaks or the appearance of newly emerging viruses (Table 1).

Interestingly, many predicted BCCs contain nucleotide/nucleoside analogs along with inhibitors of pyrimidine/purine biosynthesis, cap analogs, or IFNs, which also target viral RNA synthesis via IFN-induced RNases. Indeed, such combinations showed synergy in experiments performed in our and other laboratories (Bellobuono et al., 1997; Byrn et al., 2015; Falloon et al., 2000; Herring et al., 2021; Ianevski et al., 2021a, 2021b; Li et al., 2020, 2022; Phillips et al., 2015; Pires de Mello et al., 2018; Schultz et al., 2022; Tong et al., 2018; Wedemeyer et al., 2013). Thus, our preliminary results suggest that scores could correlate with the antiviral efficacy of BCCs and that some of these combinations could be used as pan- and even cross-virus family .

### Limitations of the study

The MoA of many BSAs remains elusive. In addition, many BSAs have been tested only *in vitro*. These lowered the final scores of BSAs and BCCs and thus affected the prediction capacity of our approach. Therefore, the MoAs of BSAs and BCCs should be studied *in vitro* and their efficacy and toxicity should be evaluated *in vivo*. In addition, immunological properties and RoA of mono- and combinational therapies should be evaluated. Finally, the prospects for clinical trials of the most effective and least toxic drug combinations should be assessed.

## Conclusions and future perspectives

New life-threatening viruses emerge and pose a serious threat to public health. Thereby, broadly effective antiviral therapies must be developed to be ready for clinical trials, which should begin soon after a new virus started to spread from human to human (Andersen et al., 2020). To identify novel pan- and cross-virus family treatments, we established a scoring system, which is based on analysis of conserved druggable virus-host interactions, MoAs, immunomodulatory properties of BSAs, RoAs, and BSA interactions with other antivirals. The system prioritizes the development of the most promising few of the thousands of potentially viable BSAs and BCCs. However, the effectiveness of the predicted BSAs and BCCs needs to be confirmed *in vitro* and *in vivo* to prepare them for clinical trials (White et al., 2021). Therefore, we will invite researchers to validate our proposed BSAs and BCCs and optimize our approach further using mathematical modeling, machine-learning, and other tools. If handled correctly, the development of the right BSAs and BCCs can have a global impact by enhancing preparedness for future viral outbreaks, filling the void between virus identification and vaccine development with life-saving countermeasures and improving the protection of the general population against emerging viral threats.

## Supplemental information

Document S1. Figure S1

Data S1. Broad-spectrum antivirals (BSAs), their targets, mechanisms of action, immunomodulatory properties, routes of delivery, BSA-containing drug combinations (BCCs), and BSA and BCC scores

## References

[bib1] Ahmed A., Felmlee D.J. (2015). Mechanisms of hepatitis C viral resistance to direct acting antivirals. Viruses.

[bib2] Aiewsakun P., Simmonds P. (2018). The genomic underpinnings of eukaryotic virus taxonomy: creating a sequence-based framework for family-level virus classification. Microbiome.

[bib3] Alijotas-Reig J., Esteve-Valverde E., Belizna C., Selva-O'Callaghan A., Pardos-Gea J., Quintana A., Mekinian A., Anunciacion-Llunell A., Miro-Mur F. (2020). Immunomodulatory therapy for the management of severe COVID-19. Beyond the anti-viral therapy: a comprehensive review. Autoimmun. Rev..

[bib4] Andersen P.I., Ianevski A., Lysvand H., Vitkauskiene A., Oksenych V., Bjoras M., Telling K., Lutsar I., Dumpis U., Irie Y. (2020). Discovery and development of safe-in-man broad-spectrum antiviral agents. Int. J. Infect. Dis..

[bib5] Andersen P.I., Krpina K., Ianevski A., Shtaida N., Jo E., Yang J., Koit S., Tenson T., Hukkanen V., Anthonsen M.W. (2019). Novel antiviral activities of obatoclax, emetine, niclosamide, brequinar, and homoharringtonine. Viruses.

[bib6] Bellobuono A., Mondazzi L., Tempini S., Silini E., Vicari F., Ideo G. (1997). Ribavirin and interferon-alpha combination therapy vs interferon-alpha alone in the retreatment of chronic hepatitis C: a randomized clinical trial. J. Viral Hepat..

[bib7] Bosl K., Ianevski A., Than T.T., Andersen P.I., Kuivanen S., Teppor M., Zusinaite E., Dumpis U., Vitkauskiene A., Cox R.J. (2019). Common nodes of virus-host interaction revealed through an integrated network analysis. Front. Immunol..

[bib8] Byrn R.A., Jones S.M., Bennett H.B., Bral C., Clark M.P., Jacobs M.D., Kwong A.D., Ledeboer M.W., Leeman J.R., McNeil C.F. (2015). Preclinical activity of VX-787, a first-in-class, orally bioavailable inhibitor of the influenza virus polymerase PB2 subunit. Antimicrob. Agents Chemother..

[bib9] Chaudhuri S., Symons J.A., Deval J. (2018). Innovation and trends in the development and approval of antiviral medicines: 1987-2017 and beyond. Antivir. Res..

[bib10] Chen S., Wang Y., Li P., Yin Y., Bijvelds M.J., de Jonge H.R., Peppelenbosch M.P., Kainov D.E., Pan Q. (2020). Drug screening identifies gemcitabine inhibiting rotavirus through alteration of pyrimidine nucleotide synthesis pathway. Antivir. Res..

[bib11] Choi Y.K. (2021). Emerging and re-emerging fatal viral diseases. Exp. Mol. Med..

[bib12] Consortium W.H.O.S.T., Pan H., Peto R., Henao-Restrepo A.M., Preziosi M.P., Sathiyamoorthy V., Abdool Karim Q., Alejandria M.M., Hernandez Garcia C., Kieny M.P. (2021). Repurposed antiviral drugs for covid-19 - interim WHO solidarity trial results. N. Engl. J. Med..

[bib13] de Buhr H., Lebbink R.J. (2018). Harnessing CRISPR to combat human viral infections. Curr. Opin. Immunol..

[bib14] De Clercq E. (2005). Emerging anti-HIV drugs. Expert. Opin. Emerg. Drugs.

[bib15] D'Elia R.V., Harrison K., Oyston P.C., Lukaszewski R.A., Clark G.C. (2013). Targeting the "cytokine storm" for therapeutic benefit. Clin. Vaccin. Immunol..

[bib16] Denisova O.V., Kakkola L., Feng L., Stenman J., Nagaraj A., Lampe J., Yadav B., Aittokallio T., Kaukinen P., Ahola T. (2012). Obatoclax, saliphenylhalamide, and gemcitabine inhibit influenza a virus infection. J. Biol. Chem..

[bib17] Denisova O.V., Soderholm S., Virtanen S., Von Schantz C., Bychkov D., Vashchinkina E., Desloovere J., Tynell J., Ikonen N., Theisen L.L. (2014). Akt inhibitor MK2206 prevents influenza pH1N1 virus infection *in vitro*. Antimicrob. Agents Chemother..

[bib18] Dyall J., Nelson E.A., DeWald L.E., Guha R., Hart B.J., Zhou H., Postnikova E., Logue J., Vargas W.M., Gross R. (2018). Identification of combinations of approved drugs with synergistic activity against Ebola virus in cell cultures. J. Infect. Dis..

[bib19] Fajgenbaum D.C., June C.H. (2020). Cytokine storm. N. Engl. J. Med..

[bib20] Falloon J., Piscitelli S., Vogel S., Sadler B., Mitsuya H., Kavlick M.F., Yoshimura K., Rogers M., LaFon S., Manion D.J. (2000). Combination therapy with amprenavir, abacavir, and efavirenz in human immunodeficiency virus (HIV)-infected patients failing a protease-inhibitor regimen: pharmacokinetic drug interactions and antiviral activity. Clin. Infect. Dis..

[bib21] Finch C.L., Dyall J., Xu S., Nelson E.A., Postnikova E., Liang J.Y., Zhou H., DeWald L.E., Thomas C.J., Wang A. (2021). Formulation, stability, pharmacokinetic, and modeling studies for tests of synergistic combinations of orally available approved drugs against Ebola virus *in vivo*. Microorganisms.

[bib22] Herring S., Oda J.M., Wagoner J., Kirchmeier D., O'Connor A., Nelson E.A., Huang Q., Liang Y., DeWald L.E., Johansen L.M. (2021). Inhibition of arenaviruses by combinations of orally available approved drugs. Antimicrob. Agents Chemother..

[bib23] Holstein S.A., McCarthy P.L. (2017). Immunomodulatory drugs in multiple myeloma: mechanisms of action and clinical experience. Drugs.

[bib24] Ianevski A., Kulesskiy E., Krpina K., Lou G., Aman Y., Bugai A., Aasumets K., Akimov Y., Bulanova D., Gildemann K. (2020). Chemical, physical and biological triggers of evolutionary conserved Bcl-xL-mediated apoptosis. Cancers (Basel).

[bib25] Ianevski A., Yao R., Biza S., Zusinaite E., Mannik A., Kivi G., Planken A., Kurg K., Tombak E.M., Ustav M. (2020). Identification and tracking of antiviral drug combinations. Viruses.

[bib26] Ianevski A., Yao R., Fenstad M.H., Biza S., Zusinaite E., Reisberg T., Lysvand H., Loseth K., Landsem V.M., Malmring J.F. (2020). Potential antiviral options against SARS-CoV-2 infection. Viruses.

[bib27] Ianevski A., Yao R., Lysvand H., Grodeland G., Legrand N., Oksenych V., Zusinaite E., Tenson T., Bjoras M., Kainov D.E. (2021). Nafamostat-Interferon-alpha combination suppresses SARS-CoV-2 infection *in vitro* and *in vivo* by cooperatively targeting host TMPRSS2. Viruses.

[bib28] Ianevski A., Yao R., Zusinaite E., Lello L.S., Wang S., Jo E., Yang J., Ravlo E., Wang W., Lysvand H. (2021). Synergistic interferon-alpha-based combinations for treatment of SARS-CoV-2 and other viral infections. Viruses.

[bib29] Ianevski A., Zusinaite E., Kuivanen S., Strand M., Lysvand H., Teppor M., Kakkola L., Paavilainen H., Laajala M., Kallio-Kokko H. (2018). Novel activities of safe-in-human broad-spectrum antiviral agents. Antivir. Res..

[bib30] Ilyas J.A., Vierling J.M. (2014). An overview of emerging therapies for the treatment of chronic hepatitis C. Med. Clin. North Am..

[bib31] Jain J., Almquist S.J., Shlyakhter D., Harding M.W. (2001). VX-497: a novel, selective IMPDH inhibitor and immunosuppressive agent. J. Pharm. Sci..

[bib32] Kakkola L., Denisova O.V., Tynell J., Viiliainen J., Ysenbaert T., Matos R.C., Nagaraj A., Ohman T., Kuivanen S., Paavilainen H. (2013). Anticancer compound ABT-263 accelerates apoptosis in virus-infected cells and imbalances cytokine production and lowers survival rates of infected mice. Cell Death Dis..

[bib33] Kaur R., Kumar K. (2021). Synthetic and medicinal perspective of quinolines as antiviral agents. Eur. J. Med. Chem..

[bib34] Kim S., Chen J., Cheng T., Gindulyte A., He J., He S., Li Q., Shoemaker B.A., Thiessen P.A., Yu B. (2021). PubChem in 2021: new data content and improved web interfaces. Nucleic Acids Res..

[bib35] Ko M., Chang S.Y., Byun S.Y., Ianevski A., Choi I., Pham Hung d'Alexandry d'Orengiani A.L., Ravlo E., Wang W., Bjoras M., Kainov D.E. (2021). Screening of FDA-approved drugs using a MERS-CoV clinical isolate from South Korea identifies potential therapeutic options for COVID-19. Viruses.

[bib36] Kuhn J.H. (2021). Virus taxonomy. Encyclopedia Virol..

[bib37] Kuivanen S., Bespalov M.M., Nandania J., Ianevski A., Velagapudi V., De Brabander J.K., Kainov D.E., Vapalahti O. (2017). Obatoclax, saliphenylhalamide and gemcitabine inhibit Zika virus infection *in vitro* and differentially affect cellular signaling, transcription and metabolism. Antivir. Res..

[bib38] Langendries L., Abdelnabi R., Neyts J., Delang L. (2021). Repurposing drugs for mayaro virus: identification of EIDD-1931, favipiravir and suramin as mayaro virus inhibitors. Microorganisms.

[bib39] Larkin M.A., Blackshields G., Brown N.P., Chenna R., McGettigan P.A., McWilliam H., Valentin F., Wallace I.M., Wilm A., Lopez R. (2007). Clustal W and clustal X version 2.0. Bioinformatics.

[bib40] Levanova A., Poranen M.M. (2018). RNA interference as a prospective tool for the control of human viral infections. Front. Microbiol..

[bib41] Li P., Li Y., Wang Y., Liu J., Lavrijsen M., Li Y., Zhang R., Verstegen Monique M.A., Wang Y., Li T.-C. (2022). Recapitulating hepatitis E virus–host interactions and facilitating antiviral drug discovery in human liver–derived organoids. Sci. Adv..

[bib42] Li W.C., Wang M.R., Kong L.B., Ren W.G., Zhang Y.G., Nan Y.M. (2011). Peginterferon alpha-based therapy for chronic hepatitis B focusing on HBsAg clearance or seroconversion: a meta-analysis of controlled clinical trials. BMC Infect. Dis..

[bib43] Li Y., Li P., Li Y., Zhang R., Yu P., Ma Z., Kainov D.E., de Man R.A., Peppelenbosch M.P., Pan Q. (2020). Drug screening identified gemcitabine inhibiting hepatitis E virus by inducing interferon-like response via activation of STAT1 phosphorylation. Antivir. Res..

[bib44] Li Y., Miao Z., Li P., Zhang R., Kainov D.E., Ma Z., de Man R.A., Peppelenbosch M.P., Pan Q. (2021). Ivermectin effectively inhibits hepatitis E virus replication, requiring the host nuclear transport protein importin alpha1. Arch. Virol..

[bib45] Lin F.C., Young H.A. (2014). Interferons: success in anti-viral immunotherapy. Cytokine Growth Factor Rev..

[bib46] Madelain V., Duthey A., Mentre F., Jacquot F., Solas C., Lacarelle B., Vallve A., Barron S., Barrot L., Mundweiler S. (2020). Ribavirin does not potentiate favipiravir antiviral activity against Ebola virus in non-human primates. Antivir. Res..

[bib47] Martin W.R., Cheng F. (2020). Repurposing of FDA-approved toremifene to treat COVID-19 by blocking the spike glycoprotein and NSP14 of SARS-CoV-2. J. Proteome Res..

[bib48] Massari S., Desantis J., Nizi M.G., Cecchetti V., Tabarrini O. (2021). Inhibition of influenza virus polymerase by interfering with its protein-protein interactions. ACS Infect. Dis..

[bib49] Monto A.S. (2006). Vaccines and antiviral drugs in pandemic preparedness. Emerg. Infect. Dis..

[bib50] Montoya M.C., Krysan D.J. (2018). Repurposing estrogen receptor antagonists for the treatment of infectious disease. mBio.

[bib51] Morris D.J. (1994). Adverse effects and drug interactions of clinical importance with antiviral drugs. Drug Saf..

[bib53] Park A., Iwasaki A. (2020). Type I and type III interferons - induction, signaling, evasion, and application to combat COVID-19. Cell Host Microbe.

[bib54] Phillips S., Chokshi S., Chatterji U., Riva A., Bobardt M., Williams R., Gallay P., Naoumov N.V. (2015). Alisporivir inhibition of hepatocyte cyclophilins reduces HBV replication and hepatitis B surface antigen production. Gastroenterology.

[bib55] Pickett B.E., Greer D.S., Zhang Y., Stewart L., Zhou L., Sun G., Gu Z., Kumar S., Zaremba S., Larsen C.N. (2012). Virus pathogen database and analysis resource (ViPR): a comprehensive bioinformatics database and analysis resource for the coronavirus research community. Viruses.

[bib56] Pires de Mello C.P., Tao X., Kim T.H., Bulitta J.B., Rodriquez J.L., Pomeroy J.J., Brown A.N. (2018). Zika virus replication is substantially inhibited by novel favipiravir and interferon alpha combination regimens. Antimicrob. Agents Chemother..

[bib57] Pizzorno A., Padey B., Terrier O., Rosa-Calatrava M. (2019). Drug repurposing approaches for the treatment of influenza viral infection: reviving old drugs to fight against a long-lived enemy. Front. Immunol..

[bib58] Pushpakom S., Iorio F., Eyers P.A., Escott K.J., Hopper S., Wells A., Doig A., Guilliams T., Latimer J., McNamee C. (2019). Drug repurposing: progress, challenges and recommendations. Nat. Rev. Drug Discov..

[bib59] Radhakrishnan M.L., Tidor B. (2008). Optimal drug cocktail design: methods for targeting molecular ensembles and insights from theoretical model systems. J. Chem. Inf. Model.

[bib60] Rogers D., Hahn M. (2010). Extended-connectivity fingerprints. J. Chem. Inf. Model.

[bib61] Salazar G., Zhang N., Fu T.M., An Z. (2017). Antibody therapies for the prevention and treatment of viral infections. NPJ Vaccin..

[bib62] Schor S., Einav S. (2018). Repurposing of kinase inhibitors as broad-spectrum antiviral drugs. DNA Cell Biol..

[bib63] Schormann N., Sommers C.I., Prichard M.N., Keith K.A., Noah J.W., Nuth M., Ricciardi R.P., Chattopadhyay D. (2011). Identification of protein-protein interaction inhibitors targeting vaccinia virus processivity factor for development of antiviral agents. Antimicrob. Agents Chemother..

[bib64] Schultz D.C., Johnson R.M., Ayyanathan K., Miller J., Whig K., Kamalia B., Dittmar M., Weston S., Hammond H.L., Dillen C. (2022). Pyrimidine inhibitors synergize with nucleoside analogues to block SARS-CoV-2. Nature.

[bib65] Shyr Z.A., Cheng Y.S., Lo D.C., Zheng W. (2021). Drug combination therapy for emerging viral diseases. Drug Discov. Today.

[bib66] Soderholm S., Kainov D.E., Ohman T., Denisova O.V., Schepens B., Kulesskiy E., Imanishi S.Y., Corthals G., Hintsanen P., Aittokallio T. (2016). Phosphoproteomics to characterize host response during influenza A virus infection of human macrophages. Mol. Cell Proteomics.

[bib67] Tong X., Smith J., Bukreyeva N., Koma T., Manning J.T., Kalkeri R., Kwong A.D., Paessler S. (2018). Merimepodib, an IMPDH inhibitor, suppresses replication of Zika virus and other emerging viral pathogens. Antivir. Res..

[bib68] Tummino T.A., Rezelj V.V., Fischer B., Fischer A., O'Meara M.J., Monel B., Vallet T., White K.M., Zhang Z., Alon A. (2021). Drug-induced phospholipidosis confounds drug repurposing for SARS-CoV-2. Science.

[bib69] Ursu O., Holmes J., Bologa C.G., Yang J.J., Mathias S.L., Stathias V., Nguyen D.T., Schurer S., Oprea T. (2019). DrugCentral 2018: an update. Nucleic Acids Res..

[bib70] Vaillant A. (2016). Nucleic acid polymers: broad spectrum antiviral activity, antiviral mechanisms and optimization for the treatment of hepatitis B and hepatitis D infection. Antivir. Res..

[bib71] Walther R., Rautio J., Zelikin A.N. (2017). Prodrugs in medicinal chemistry and enzyme prodrug therapies. Adv. Drug Deliv. Rev..

[bib72] Wang B., Zeng H., Han Y. (2020). Random walks in time-varying networks with memory. Phys. Rev. E..

[bib73] Wedemeyer H., Jensen D., Herring R., Ferenci P., Ma M.M., Zeuzem S., Rodriguez-Torres M., Bzowej N., Pockros P., Vierling J. (2013). PROPEL: a randomized trial of mericitabine plus peginterferon alpha-2a/ribavirin therapy in treatment-naive HCV genotype 1/4 patients. Hepatology.

[bib74] White J.M., Schiffer J.T., Bender Ignacio R.A., Xu S., Kainov D., Ianevski A., Aittokallio T., Frieman M., Olinger G.G., Polyak S.J. (2021). Drug combinations as a first line of defense against coronaviruses and other emerging viruses. mBio.

[bib75] Wishart D.S., Feunang Y.D., Guo A.C., Lo E.J., Marcu A., Grant J.R., Sajed T., Johnson D., Li C., Sayeeda Z. (2018). DrugBank 5.0: a major update to the DrugBank database for 2018. Nucleic Acids Res..

[bib52] World Health Organization (2018). https://apps.who.int/iris/handle/10665/272442.

[bib76] Yang J., Konig A., Park S., Jo E., Sung P.S., Yoon S.K., Zusinaite E., Kainov D., Shum D., Windisch M.P. (2021). A new high-content screening assay of the entire hepatitis B virus life cycle identifies novel antivirals. JHEP Rep..

[bib77] Yin W., Luan X., Li Z., Zhou Z., Wang Q., Gao M., Wang X., Zhou F., Shi J., You E. (2021). Structural basis for inhibition of the SARS-CoV-2 RNA polymerase by suramin. Nat. Struct. Mol. Biol..

[bib78] Zhao Y., Ren J., Harlos K., Jones D.M., Zeltina A., Bowden T.A., Padilla-Parra S., Fry E.E., Stuart D.I. (2016). Toremifene interacts with and destabilizes the Ebola virus glycoprotein. Nature.

[bib79] Zoulim F. (2005). Combination of nucleoside analogues in the treatment of chronic hepatitis B virus infection: lesson from experimental models. J. Antimicrob. Chemother.

[bib80] Zusinaite E., Ianevski A., Niukkanen D., Poranen M.M., Bjoras M., Afset J.E., Tenson T., Velagapudi V., Merits A., Kainov D.E. (2018). A systems approach to study immuno- and neuro-modulatory properties of antiviral agents. Viruses.

