# Supplementary material for: Mono- and combinational drug therapies for global viral pandemic preparedness
Source: iScience. 2022 Mar 17;25(4):104112. doi: 10.1016/j.isci.2022.104112 (PMC8983340; doi:10.1016/j.isci.2022.104112)
Supplement: Document S1. Figure S1 [file mmc1.pdf]

iScience, Volume *25*

Supplemental information

Mono- and combinational drug therapies

for global viral pandemic preparedness

Aleksandr Ianevski, Rouan Yao, Ronja M. Simonsen, Vegard Myhre, Erlend Ravlo, Gerda D. Kaynova, Eva Zusinaite, Judith M. White, Stephen J. Polyak, Valentyn Oksenych, Marc P. Windisch, Qiuwei Pan, Eglė Lastauskienė, Astra Vitkauskienė, Algimantas Matukevičius, Tanel Tenson, Magnar Bjørås, and Denis E. Kainov

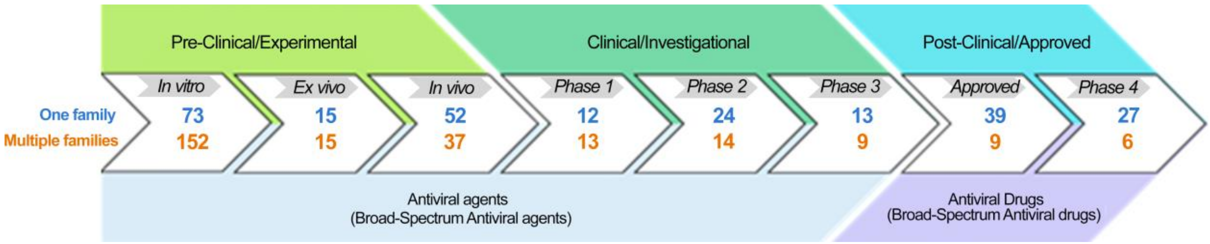

Fig. S1. BSAs and stages of their development. BSAs that have reached the developmental stage in question for at least one viral indication are shown in blue, whereas BSAs that have reached the developmental stage in question for at least two viral indications are shown in orange.
